# AMR in wild animals and plants: global trends and future priorities in wildlife-associated antimicrobial resistance research

**DOI:** 10.1038/s44259-026-00226-3

**Published:** 2026-06-02

**Authors:** Holly J. Tipper, Rachel A. Payne, Isobel C. Stanton, Jennifer M. G. Shelton, Alwyn Hart, Wiebke Schmidt, Purvi Mali, Andrew C. Singer, Daniel S. Read

**Affiliations:** 1 UK Centre for Ecology & Hydrology (UKCEH), Wallingford, OX10 8BB Oxfordshire UK; 2 Formerly Chief Scientist’s Group, Environment Agency, Horizon House, Deanery Road, Bristol, BS1 5AH UK; 3 Chief Scientist’s Group, Environment Agency, Horizon House, Deanery Road, Bristol, BS1 5AH UK

**Keywords:** Ecology, Ecology, Zoology

## Abstract

Antimicrobial resistance (AMR) is a major global health concern, involving complex transmission pathways linking humans, domestic animals, wildlife, and the environment. Wild animals and plants can harbour and transmit AMR, while also serving as sentinels. Here, we aimed to evaluate current knowledge and research gaps on AMR in wild animals and plants to inform One Health research and policy. We conducted a semi‑systematic review of AMR in wild animals and plants, generating a dataset of 866 publications and analysing metadata on host taxa, microbial and genetic targets, and analytical approaches. The literature shows strong taxonomic, geographic, and methodological biases, with mammals and birds dominating, whereas plants (*n* = 14) and amphibians (*n* = 10) were rarely studied. Resistant fungi were also under‑represented (2% of studies), while *Escherichia* spp. accounted for 33% of microbial targets. Employing wildlife‑based surveillance offers a useful policy tool to address key AMR gaps at human-animal-environment interfaces.

## Introduction

Antimicrobial resistance (AMR) is one of the most serious global health threats facing society, with bacterial AMR alone predicted to have been responsible for approximately 1.27 million deaths in 2019^1^. AMR exists in clinical, veterinary, agricultural, and environmental settings, and as a result, requires a holistic One Health approach to address it^2^.

AMR has existed naturally in the environment for millennia due to microbial competition for resources^3^. However, anthropogenic activities can elevate AMR above natural background levels. This includes the release of resistant microbes and AMR-driving chemicals (e.g., antimicrobials, metals, and biocides) from wastewater and agriculture^4,5^. It has also been shown that clinically relevant AMR can emerge from environmental origins^6^, potentially contributing to negative human health outcomes. Acquiring such data and linking environmental AMR to human health outcomes is challenging; risk assessment and mitigation strategies would benefit from considerably more research to understand environmental transmission from a more mechanistic (empirical) perspective^7^.

Although environmental AMR has historically been overlooked, it has become a rapidly growing research area over the last decade, with calls from researchers for additional studies^8^. Water, soils and sediments have frequently been used to investigate environmental reservoirs and receptors of AMR^4,9,10^. However, such environmental matrices can be prone to significant biological and chemical fluctuations over short spatial and temporal extents^11^. Evidence suggests that the microbiomes of wild animals and plants may respond to and reflect environmental pressures^12^, with intermittent or chronic exposure to pollution potentially resulting in temporary or permanent changes in microbial communities. Wildlife microbiomes can harbour elevated levels of mixtures of disease-causing microorganisms and antimicrobial resistance gene (ARG)-harbouring microorganisms. As such, wild animals and wild plants could also represent a transmission pathway between anthropogenic and environmental compartments, either as food sources or by facilitating the movement of AMR between hosts (e.g., humans, livestock, wildlife)^13^. In addition, wildlife microbiomes may act as hotspots for horizontal gene transfer (HGT), serving as sinks for ARGs and mixing pools for the creation of novel variants^14–16^—a potentially overlooked dimension impacting clinical AMR^17,18^.

The use of wildlife as an indicator of ecosystem health is widely established and is undertaken internationally by environmental regulators^19,20^. This highlights their potential as tools for monitoring environmental AMR, potentially reducing some of the complexity involved in direct sampling of environmental matrices. Some studies have explicitly suggested the use of certain species as sentinels for AMR monitoring, for example, bivalve molluscs^21^ and small mammals^22^. However, given that our understanding of AMR dynamics in wildlife is limited, it is unclear which organisms and ecological and trophic characteristics are important when selecting sentinel targets. Furthermore, the choice of species, method and endpoint for surveillance efforts often depends on the research question being asked.

Previous reviews have explored specific aspects of the literature on AMR in wildlife. For instance, Zeballos-Gross et al. (2021) conducted a scoping review to assess the role of gulls as potential reservoirs of AMR^23^. Vittecoq et al. (2016) examined various dimensions of AMR in wildlife, focusing specifically on antibacterial resistance in animal hosts^24^. Similarly, Ramey and Ahlstrom (2020) critically reviewed the literature, focusing on antibiotic-resistant bacteria in wild, zoo and peridomestic animals, and called for hypothesis-driven research^25^. Torres et al. (2020) analysed spatial and temporal trends in studies from a single database, summarising target bacteria and host species^26^, while Li et al. (2024) reviewed clinically relevant resistance in wild animals^27^. Greig et al. (2014) delivered a scoping review on the role of wildlife in transmission of AMR and/or bacterial pathogens to the food chain^28^. Finally, Doyle et al. (2025) highlighted factors contributing to the dissemination of AMR in wildlife and to its transmission in the environment through wildlife interactions, as well as key species used in wildlife monitoring and potential knowledge gaps in this field^16^. Building on these foundational studies, we conducted a holistic, semi-systematic review of the literature to reveal the current state of knowledge on AMR, including bacterial and fungal AMR, in both animal and plant wildlife. A semi‑systematic review was selected to enable transparent synthesis of key themes and evidence gaps within a heterogeneous and evolving literature, allowing methodological flexibility where systematic review conventions would be unnecessarily restrictive for the review’s purpose. To structure the review, the following research question was formulated: what is the current landscape of published research on AMR in wild animals and plants, and where are the research gaps? Unlike previous reviews, we include wild plants because humans, animals, and crops may encounter them through foraging, medicinal use, ingestion, or shared habitats, posing potential exposure pathways. This work is an expansion of work by the authors previously published as an English Environment Agency report^29^, with updated searches and expanded search terms, resulting in greater inclusion of publications. The findings of this review will enable a comprehensive assessment of the environments, host species, microbial targets, and laboratory analytical methods represented in the current literature. We aimed to identify key areas where further research is needed to advance our understanding of AMR within a One Health framework. Additionally, we aimed to highlight potential targets for environmental AMR surveillance, to offer critical insights needed for guiding both future research and policy development.

## Results

### Description of review process

**Fig. 1:**
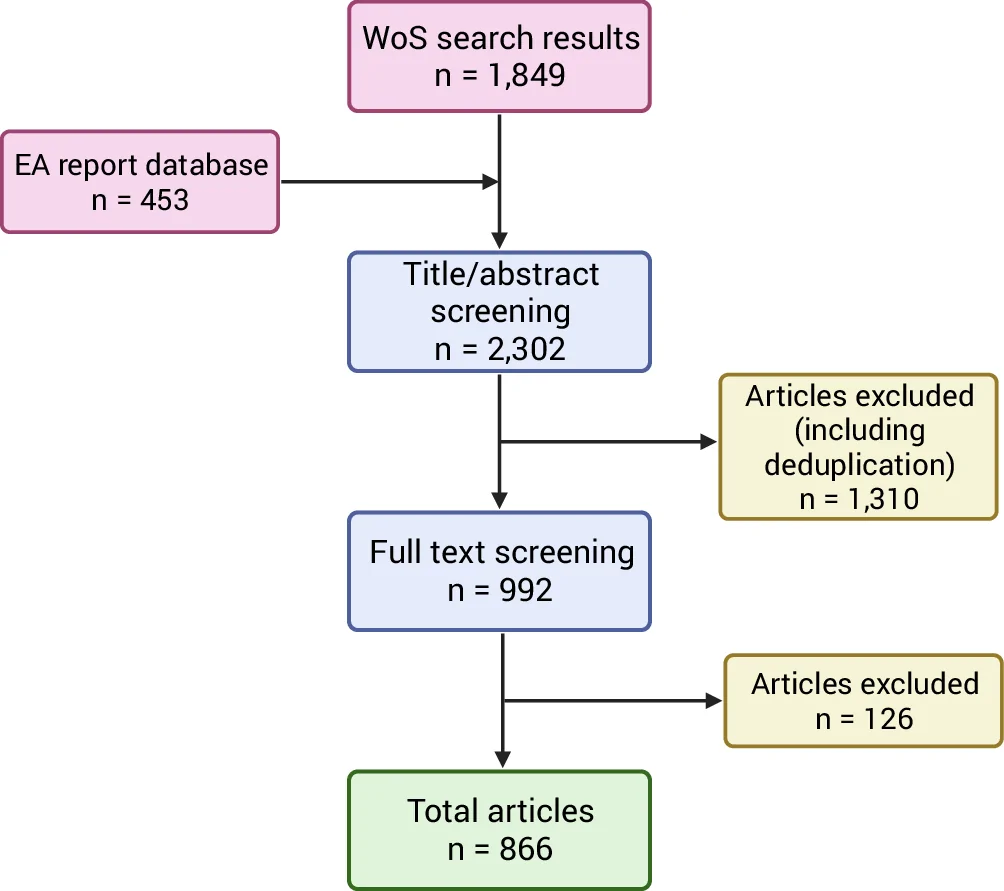
Semi-systematic review process used here. Flow diagram representing the semi-systematic review process applied in this study, including literature identification, screening, and final inclusions.

Web of Science (WoS) searches (as described in the Methods) yielded a total of 1849 publications, which once combined with 453 publications from the Environment Agency report database^29^, resulted in screening of 2302 titles and abstracts (Fig. 1). Of these, 992 publications were eligible for full text screening. The final publication database comprised 866 publications that were included in the following analyses. The database can be found as a Supplementary File.

### Spatiotemporal distribution of publications

**Fig. 2:**
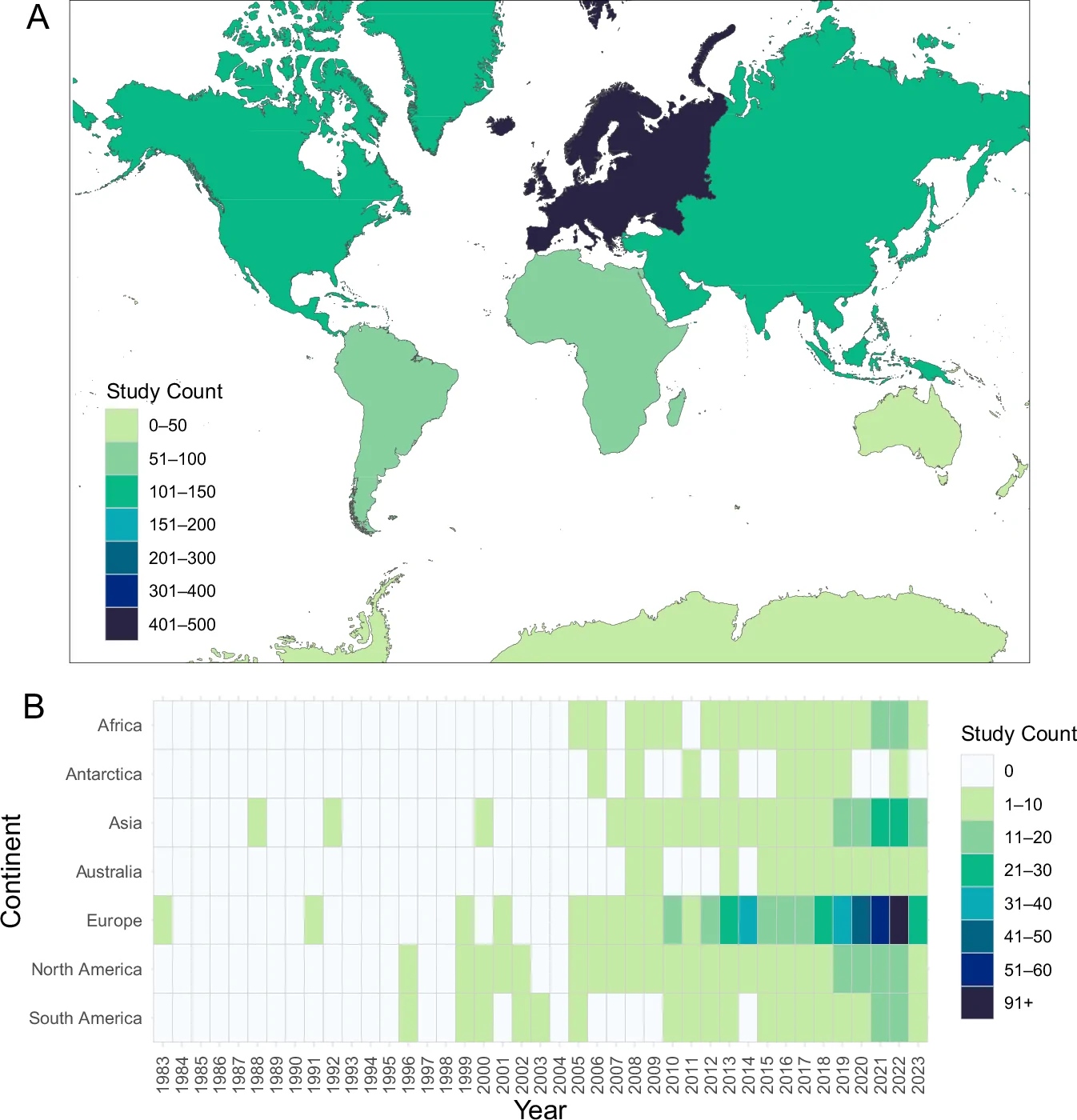
Spatiotemporal distribution of publications investigating AMR in wild animals and plants. **A** The number of publications investigating AMR in wild animals and plants per continent of study: Africa (*n* = 86), Antarctica (*n* = 10), Asia (*n* = 139), Oceania (*n* = 36), Europe (*n* = 444), North America (*n* = 141), and South America (*n* = 90). **B** Temporal spread of publications investigating AMR in wild animals per continent (1983–2023).

Spatial analysis was conducted to understand whether there was over- or under-representation of literature from certain countries. This also indicates over/under-representation of host species based on their global distribution. The top five most prolific countries of study were Spain (number of publications = 87), the USA (*n* = 86), Italy (*n* = 61), Portugal (*n* = 59) and Brazil (*n* = 52). The number of publications per continent were as follows: Europe (*n* = 444), North America (*n* = 141), Asia (*n* = 139), South America (*n* = 90), Africa (*n* = 86), Oceania (*n* = 36), and Antarctica (*n* = 10) (Fig. 2a). Some countries show elevated publication counts, which may be influenced by the strong productivity of some research teams. In some cases, the same samples were studied in multiple publications, causing a higher representation of these countries of research in the database.

The habitat types identified in the literature were: terrestrial environments (*n* = 676), coastal environments (*n* = 164), freshwater environments (*n* = 147) and marine environments (*n* = 51). Nine publications had an unknown habitat type (i.e., when the target host taxa were unknown or lacked sufficient detail) (Fig. S2).

Publication years ranged from 1983 to 2023, with a general upwards trend in publication numbers over time and over 50% of publications occurring since 2019 (Fig. 2b). It should be noted that the cut-off date for publications to be included in the database was August 2023, therefore the values for 2023 may appear lower than the actual total publications for this year. This recent increase in publications relating to AMR in wild animals and plants reflects the growing interest in environmental AMR in a One Health context.

### Targeted host taxa

**Fig. 3:**
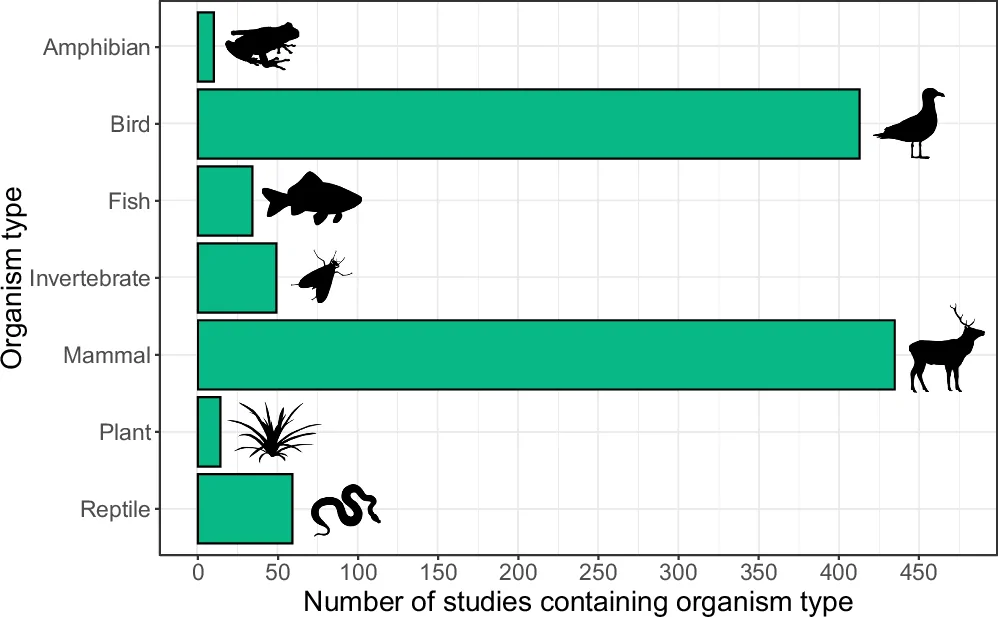
Number of publications investigating AMR in wild animals and plants. Six publications did not specify the target organism further than ‘wildlife’, so the organisms studied were marked as ‘unknown’ in the database and are not represented in the figure.

Each publication included in the analysis was categorised based on high-level taxonomic groups, depending on the target host taxa. These broad groups included amphibians, birds, fish, invertebrates, mammals, plants and reptiles. The most targeted host taxa were mammals and birds (Fig. 3). Of the 866 publications in the database, 435 targeted mammals, 413 targeted birds, 59 targeted reptiles, 49 targeted invertebrates, 34 targeted fish, 14 targeted plants and 10 targeted amphibians. Six publications did not specify the target organism further than ‘wildlife’, so the organisms studied were marked as ‘unknown’ in the database.

In this database, each new record represents an “occurrence,” which is defined at the level of the specific metric being analysed. While publications are counted once, occurrences capture every unique combination of attributes recorded. For example, if a single study reports multiple bird species, each species becomes a separate occurrence for the host taxa metric. Similarly, if the same species is analysed for multiple target organisms, genes, or analysis types, each of these is counted as a distinct occurrence for those respective metrics. However, multiple individuals of the same species within one publication are still treated as a single host occurrence. After generating occurrences for each metric, the data were then consolidated by publication to allow comparative analyses across studies. This layered approach ensures that the database reflects the full complexity of study designs across different dimensions (see Methods for more details).

**Fig. 4:**
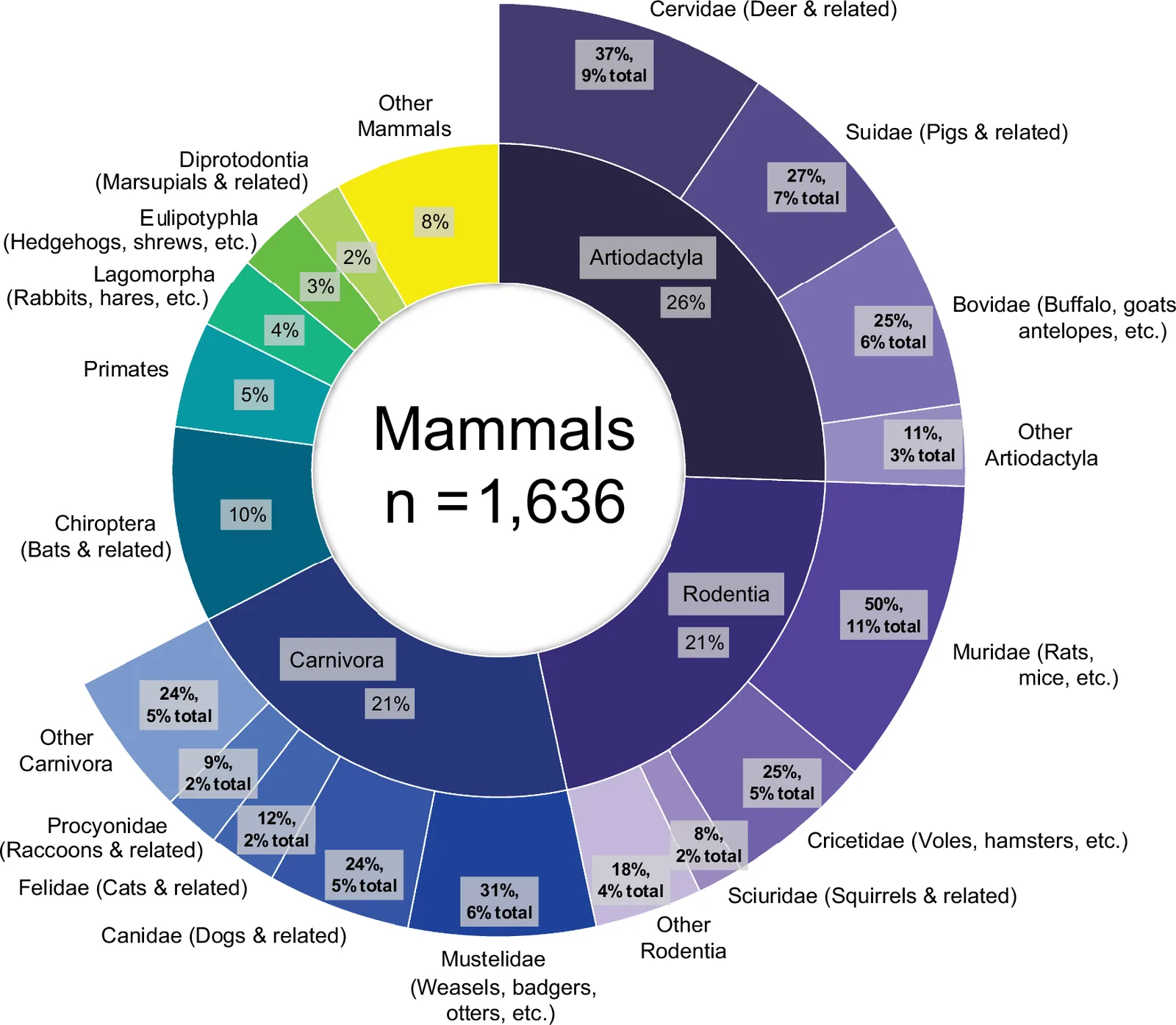
Proportion of different types of mammal taxa (number of specific occurrences = 1636; number of publications = 435). Inside rings represent taxa at order level, whereas outside rings indicate lower taxonomic levels, i.e., family, superfamily or suborder. Common names shown in parentheses indicate examples.

Within the 435 mammal publications, there were 1,636 specific mammal occurrences (Fig. 4). Of the mammals, the order Artiodactyla (even-toed ungulates) was sampled most frequently (number of occurrences = 418) (e.g.^30–32^,). The most studied families within Artiodactyla were *Cervidae* (e.g., deer and relatives) (*n* = 154), *Suidae* (e.g., pigs and relatives) (*n* = 112) and *Bovidae* (e.g., cattle) (*n* = 106). Notably, the wild boar (an ungulate in the *Suidae* family) was the most studied species in the database, with 100 occurrences (e.g. refs. ^33–35^,). As previously mentioned, certain study metrics, such as host taxa (e.g., wild boar and deer) and country of study are likely influenced by the strong productivity of some research teams. Mammals within the order Rodentia were the second most studied throughout the literature (e.g. refs. ^36,37^,), with *Muridae* (e.g., mice, rats and gerbils) (*n* = 174) and *Cricetidae* (e.g., hamsters, voles and muskrats) (*n* = 84) the most studied families. Carnivora (e.g., dogs, cats, bears, raccoons etc.) were the third most studied order (*n* = 340) (e.g.^38,39^,).

**Fig. 5:**
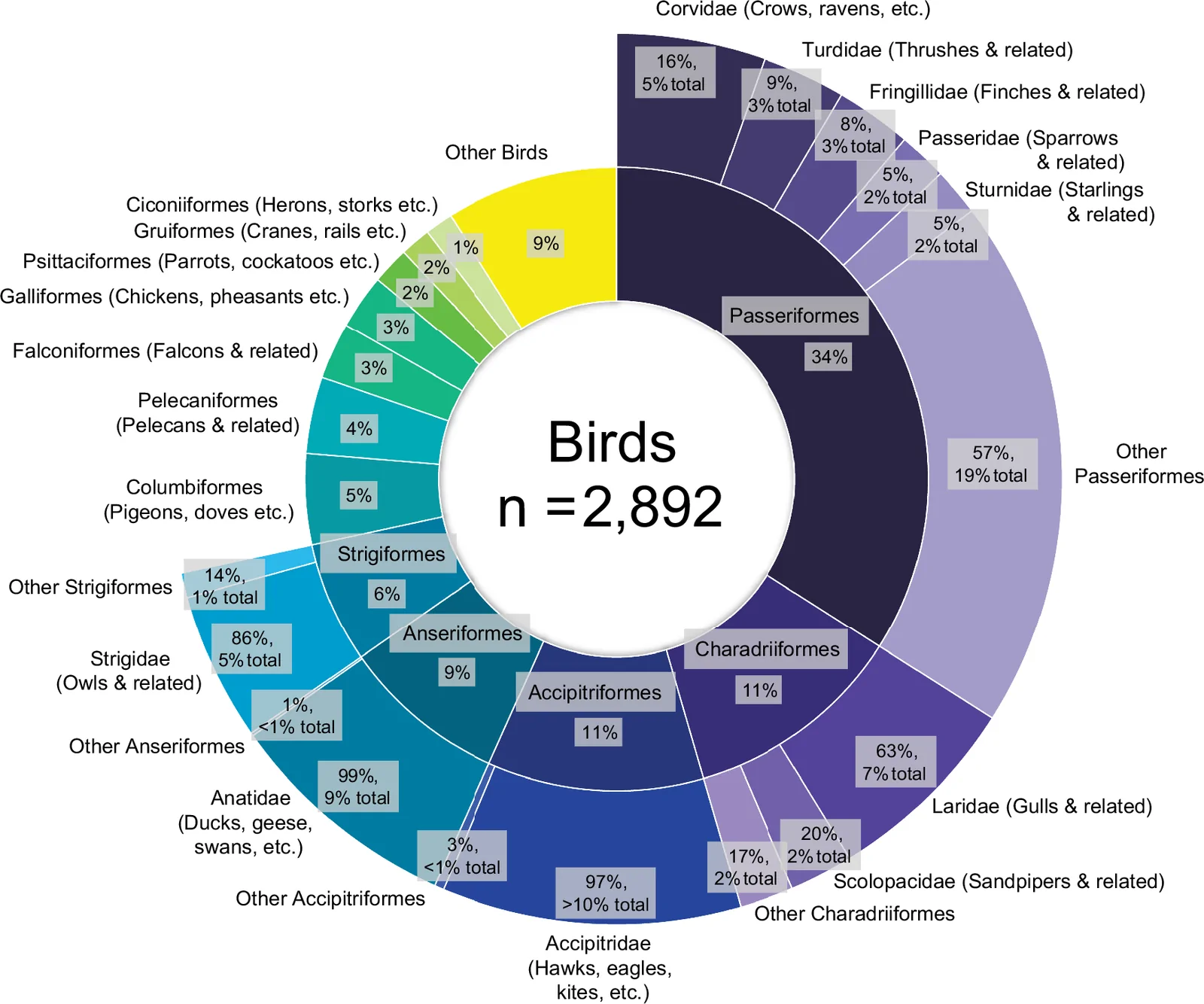
Proportion of different types of bird taxa (number of specific occurrences = 2892; number of publications = 413). Inside rings represent taxa at order level, whereas outside rings indicate lower taxonomic levels, i.e., family, superfamily or suborder. Common names shown in parentheses indicate examples.

Within the 413 bird publications, there were 2892 specific bird occurrences (Fig. 5). Just over one third of the birds occurring in the literature were of the order Passeriformes (passerines, considered the “perching birds”) (number of occurrences = 984), which contains over half of all bird species and is the most species-rich order^40,41^ (e.g., sparrows, finches, thrushes, and corvids). Of the passerines studied, the most targeted host taxa were those in the *Corvidae* family (e.g., crows, rooks, magpies, and ravens) (*n* = 156) (e.g.^42–44^,). The second most well-studied order was Charadriiformes (e.g., gulls and auks) (*n* = 332) (e.g.^45–47^,), with the most studied family *Laridae* (e.g., gulls, turns, noddies and related) (*n* = 208). The third most studied order was Accipitriformes (e.g., hawks, eagles, vultures and kites), with the majority of these (97%, *n* = 314) of these being in the family *Accipitridae*, which contains hawks, eagles, kites and related (e.g.^48,49^,).

Amphibians, reptiles, invertebrates, and fish were all less targeted host taxa than mammals and birds (Fig. 3). Following mammals and birds, reptiles were the next most targeted host taxa (e.g.^50–52^,), then invertebrates (e.g. refs. ^53–55^,), fish (e.g. refs. ^56–58^,), plants (e.g. refs. ^59–61^,) and amphibians (e.g. refs. ^62–64^,). The most targeted host taxa for the remaining organism types were as follows: for fish—the order Perciformes (perch-like fish) and Cypriniformes (carp-like fish) (19 occurrences each in the database), for reptiles—the order Squamata (e.g., lizards and snakes) (number of occurrences = 124), for invertebrates—the order Diptera (e.g., flies) (*n* = 39), and for amphibians—the order Anurans (e.g., frogs and toads) (*n* = 7). Overall, whilst the number of publications with lesser-targeted host taxa was lower, some specific taxonomic groups had relatively high occurrences (e.g., the orders Squamata and Diptera) (see Supplementary Fig S1).

There was a noticeable lack of publications focusing on AMR in wild plants (*n* = 14) (Fig. 3). Publications that sampled plants targeted grasses, legumes, conifers, woody landscape plants, eucalyptus trees and forest plants (see Supplementary Fig S1). Notably, one study included in the database aimed to use the conifer needle phyllosphere (portion of plant above ground) as a passive sampler of bioaerosolised ARGs^65^.

### Analytical methods used

Details on AMR type, culture and/or molecular-based approach, AMR methods used (e.g., antibiotic/antifungal susceptibility testing (AST), polymerase chain reaction (PCR), etc.), microorganism targeted (culture target) and ARG/other gene of choice (molecular target), were extracted from publications to facilitate an evaluation of the methods used and AMR targets focused on in wildlife.

**Fig. 6:**
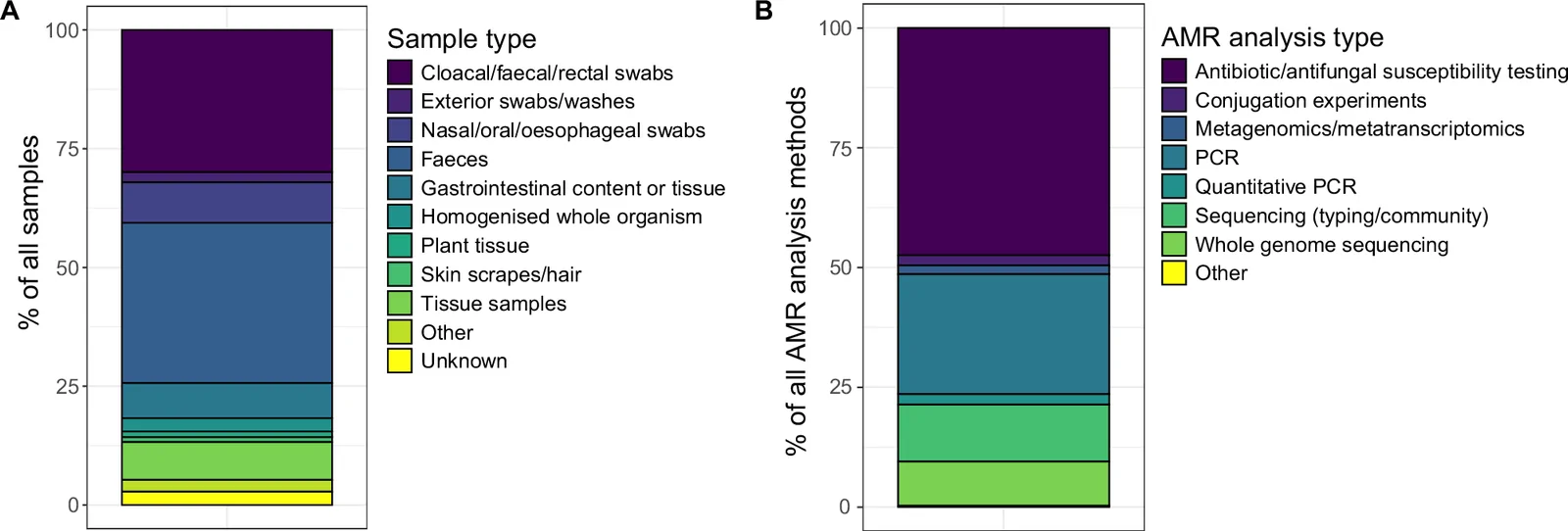
Sampling and analytical methods used in the literature investigating AMR in wild animals and plants. **A** Proportion of different sampling methods used (number of occurrences = 1080). **B** Proportion of different AMR analytical methods used (*n* = 1611).

The most frequently collected samples were cloacal/rectal/faecal swabs (e.g. refs. ^66–68^,), and faeces from animals (e.g. refs. ^69–71^,) (30% and 34% of sampling methods, respectively) (Fig. 6a). Of the very few studies focusing on AMR in wild plants, sample types included plant tissues such as fruits or tree tissue (e.g. refs. ^60,61^,).

Most publications in the database (61%) used both culture- and molecular-based approaches. Studies that used both culture- and molecular-based methods frequently isolated bacteria (often *Escherichia coli*—see section below) and performed AST on the isolates, extracted DNA and performed PCRs for ARGs. Overall, the most used analytical method was AST (Fig. 6b), which looks at phenotypic resistance traits of cultured isolates^72^ (e.g. refs. ^73–75^,). The second most used analytical method was PCR, which investigates the occurrence of resistance genes among samples using a presence/absence-based screen of resistance genes (e.g. refs. ^76–78^,). Other, less commonly used, analytical methods were qPCR, which quantifies changes in abundance of specific gene targets across samples or sites (e.g. refs. ^65,79,80^,), metagenomics/metatranscriptomics, which provide a non-targeted characterisation of the resistome of a sample (e.g. refs. ^81–83^,), and other sequencing approaches, such as whole genome sequencing, which determines the complete genome of a microorganism to enable high‑resolution analysis of its identity, evolution, and functional characteristics (e.g. refs. ^21,84,85^,).

### Target microorganisms and resistance genes

**Fig. 7:**
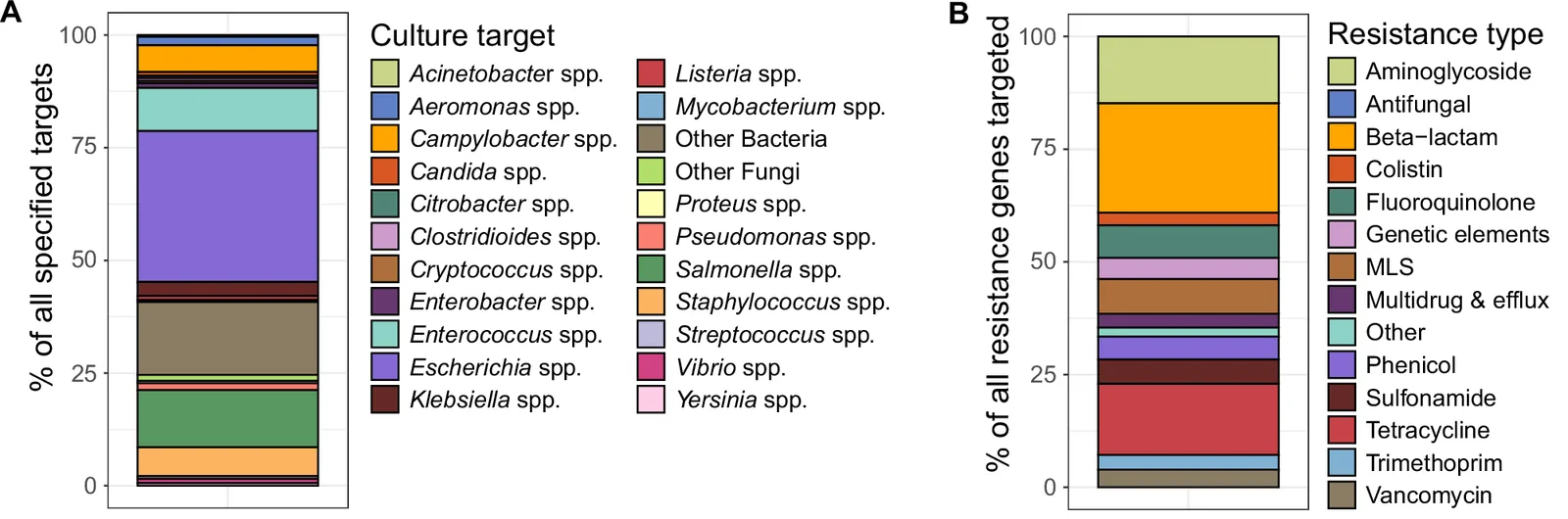
Culture targets and resistance gene targets in the literature investigating AMR in wild animals and plants. **A** Proportion of different culture targets (number of occurrences = 971). **B** Proportion of different resistant types for gene targets (*n* = 4825) in studies found in the literature. MLS = Macrolide, lincosamide and streptogramin.

Of the 866 publications collated in the database, the vast majority (98%) focused on bacteria and antibiotic resistance. Only 2% of publications (*n* = 19) investigated resistant fungi in wild flora and fauna. The sampling method often dictated the most targeted microorganism(s). For example, enteric bacteria were frequently isolated from faeces to identify common pathogens (e.g., *Salmonella* spp^86^. and *E. coli*^87^). The most targeted microorganism in culture was *Escherichia* spp., with a total of 325 occurrences in the database (33%) (Fig. 7a). This was followed by other *Enterobacterales*, such as *Enterococcus* spp. and *Salmonella* spp., as well as unspecified enteric bacteria (Fig. 7a). Other specific microorganism targets included human pathogens, such as *Campylobacter* spp.*, Pseudomonas* spp. and *Staphylococcus* spp. Of the fungi targeted, *Candida* spp. were the most common (Fig. 7a) (e.g. refs. ^88,89^,). Around 24% of all resistance genes targeted in the literature were those conferring resistance to beta-lactam antibiotics (1170 occurrences in the database) (Fig. 7b). The second most targeted resistance genes conferred resistance to tetracycline antibiotics (761 occurrences), and the third to aminoglycosides (715 occurrences). The top five most targeted genes were *bla*_*TEM*_ (174 occurrences), *tetA* (136 occurrences), *bla*_*OXA*_ (133 occurrences), *bla*_*SHV*_ (121 occurrences), and *tetB* (115 occurrences). However, the sum of all *bla*_*CTX-M*_ variants was 185 occurrences in the literature database, making it the most frequently targeted resistance gene complex (e.g. refs. ^90,91^,).

### Rationale for surveillance

The rationale provided by the authors of the studies included in the literature database was also recorded; however, at times, the rationale was unclear. If the rationale was unclear or did not fit into the main categories mentioned here, the rationale of the publication was recorded as “Other”. The most common rationale for conducting research on AMR in wildlife was to investigate the dissemination of AMR throughout the environment, followed by spillover from anthropogenic sources, and then human health/exposure to AMR in the environment. Many publications were investigating AMR in wildlife for several different reasons, for example, linking between spillover to the environment from anthropogenic contact, dissemination of AMR in the environment, and concerns towards human exposure to AMR. Rationales were less focused on explicit links to testing new methodologies. Notably, some publications testing new methods investigated using certain organisms as proxies for measuring environmental AMR (e.g., fish^92^ or bivalve molluscs^21^).

## Discussion

This analysis provides a summary of the global research landscape encompassing AMR in wild animals and plants. The trends observed here, across the most frequently targeted host taxa and microbial organisms, as well as in methodological choices such as analytical techniques and sample types, closely align with those reported in the Environment Agency study that this work substantially builds upon^29^. The literature review was conducted systematically using the WoS database, one of the largest and most comprehensive bibliometric resources^93^. Only articles published or translated into English were included. Given the breadth of journals indexed in WoS, the resulting dataset is likely broadly representative of global research. A very small number of paywalled articles could not be accessed and are therefore absent. Some bias may also reflect repeated use of the same samples across publications, potentially increasing the visibility of certain countries, host taxa, or environments; however, such repetition can also provide complementary insights into different resistance endpoints. The findings of this review identify under-researched areas of environmental AMR related to wild organisms and highlight potential taxonomic targets and methodological endpoints of interest for the environmental surveillance of AMR.

The growing body of literature on AMR in wild animals and plants reflects a rising interest in this field, suggesting that research activity, and likely funding, is on an upward trajectory. However, this literature also reveals geographic biases, with a disproportionate number of studies conducted in Europe. The methodology of this review may have influenced the findings, particularly as only English-language publications were included. This likely resulted in an overrepresentation of research from Europe and English-speaking regions, potentially overlooking significant studies published in other languages and underestimating AMR dynamics elsewhere. Future research should expand on this database, and incorporate non-English studies and broaden database searches to better capture global research activity and host diversity. Also, most studies focused on terrestrial habitats, with far fewer investigating coastal, freshwater, or marine environments, highlighting substantial habitat‑based biases in the wildlife AMR literature. Such imbalances may obscure the global dynamics of AMR, which are shaped by diverse host species, climatic conditions and anthropogenic activities, including land use practices, human-wild animal/plant interactions, use of antibiotics in human health and agricultural production, and the management of contaminated wastes. To develop a more comprehensive understanding of host species as indicators for AMR surveillance, we recommend that future research focus on filling geographic gaps and encompassing a broad range of host taxa across all continents. A total of 866 relevant publications were identified globally using the search string developed in this study, each explicitly reporting on the presence, prevalence or composition of AMR in wildlife. This indicates that an integrated One Health approach is increasingly being adopted to study AMR, reflecting a shift towards more holistic and interdisciplinary frameworks. One notable trend across these studies was the apparent bias in many study metrics, such as targeted host taxa, analytical method used, and target microorganisms or genes. We acknowledge that our review is limited by the reporting practices of the included studies, as our analysis depended on the information available in their methods and results, regardless of whether AMR was detected. Studies failing to detect AMR in wildlife may not have been published or included in databases, introducing a potential bias towards positive findings. This limitation should be considered when interpreting our results. However, a bias was evident in the relatively large number of studies focusing on mammals and birds compared to other targeted host taxa, with mammals and birds each having twice as many publications as all other organism types combined, and mammals, for example, being 31 and 43 times more likely to be targeted than plants or amphibians, respectively. It remains unclear whether this pattern accurately represents the true distribution of taxa. The focus on mammals in the research may be attributable to their genetic relatedness and shared evolutionary history with humans, their propensity to carry similar pathogens and suffer from comparable diseases, and their frequent proximity to urban centres and anthropogenic activities. Additionally, mammals are relatively accessible for sampling and capture, due to their presence in urban environments and even human households^13^. Their widespread distribution, large population sizes, and capacity to disseminate and transfer AMR to humans—via direct contact, food sources, companion animals, and environmental and household matrices^15^—position them as priority species to target for AMR surveillance. Among mammals, Artiodactyla was the most targeted order, followed by Rodentia and Carnivora. The emphasis on ungulates is likely due to their known interactions with humans through contact with livestock and hunting, making them desirable taxa for AMR surveillance. Some Carnivora, for example, badgers (Mustelidae) and foxes (Canidae), also overlap with livestock, whereas rodents are known reservoirs of many zoonotic diseases^94^. Bias in mammal-focused studies was amplified by repeated analyses of the same samples using different methods, leading to overrepresentation in the dataset. While detailed sample analysis is valuable, duplicate publications can distort key metrics, including host taxa, microbial targets, and resistance genes.

The high frequency of studies focused on birds likely reflects their wide migratory ranges, and similar to mammals, large population sizes, and frequent presence in urban centres and areas of intense anthropogenic activity, such as agricultural lands, landfills, and wastewater treatment sites. Despite more publications examining mammals overall, the number of bird occurrences was much higher. Corvids (e.g., crows, ravens, and rooks) were highly represented within the most studied bird order, Passeriformes, accounting for 16% (*n* = 156) of all passerine occurrences. As important hosts of zoonoses, corvids have been used to predict human disease outbreaks (e.g., West Nile Virus)^95^. This established use in surveillance (primarily via carcasses) and known carriage of human pathogens, suggest they may be valuable tools for monitoring environmental AMR. Birds in the order Charadriiformes, particularly gulls and their relatives (suborder Lari), were frequently studied, likely due to their ubiquity and close association with human environments and activity^16^. Similar to rodents, gulls inhabit a wide range of habitats—including coastal, inland, farmland, and urban areas—and are known to frequent landfills and wastewater treatment sites^96^. Their regular presence in human-influenced environments makes them valuable indicators for assessing AMR spillover from anthropogenic sources into wildlife. Waterfowl, such as the order Anseriformes (e.g., ducks, geese, and swans), also interact with aquatic environments, which can often act as sinks for agricultural runoff and wastewater, thereby providing transmission routes for resistant microorganisms^97^. Consequently, birds inhabiting these ecosystems are potential reservoirs and transmission routes for AMR, making them important candidates for environmental surveillance. In addition to these aquatic and urban associations, many bird species interact directly with livestock production systems, creating further opportunities for AMR transfer. Birds such as crows, doves, and thrushes frequently forage around cattle and their housing^98^, where they may acquire resistant bacteria originating from farmed animals. Their movements between farms could disperse AMR‑contaminated material across agricultural landscapes, reinforcing the role of birds as intermediaries linking wildlife, livestock, and human‑dominated environments. Accipitriformes (e.g., hawks and eagles) was the third most studied order of birds, likely due to their ecological role as apex predators. These carnivorous and opportunistic feeders consume a wide range of prey, including birds, rodents, reptiles, and insects^99^, and may also scavenge livestock carcasses, potentially exposed to antibiotics. Further, in some countries, birds of prey frequently use supplemental feeding stations stocked with livestock carcasses; for example, in Spain, vultures visit stations stocked with pig carcasses^100^. Their position as apex predators allows them to integrate AMR signals across diverse species, habitats and trophic levels, making them valuable candidates for environmental AMR surveillance, particularly when compared to herbivores and other lower trophic order animals. Overall, such interactions between wild animals and urbanised and agricultural systems represent a critical conduit for AMR transmission, emphasising the importance of enhanced One Health surveillance and intervention at this interface to mitigate broader ecological and public‑health risks.

The literature reveals a clear bias toward mammals and birds, with amphibians, fish, invertebrates, and reptiles receiving comparatively less attention. Despite some species in the latter groups being widespread and cohabiting with humans, their use in AMR surveillance may be limited by challenges in sample collection and processing. Some amphibians and reptiles may be rarer in ecological terms^101,102^, and their cryptic behaviour, low detectability, and historically limited sampling effort^103,104^, further contribute to their under‑representation in wildlife surveillance datasets compared with birds and mammals.

Additionally, faecal sampling, common in mammals and birds, is more difficult in fish, invertebrates, amphibians and reptiles. Although destructive sampling (e.g., whole insect homogenisation) can overcome some of these limitations, it raises ethical concerns compared to less invasive methods, such as catch-and-release or opportunistic faecal sampling. Furthermore, smaller sample volumes may hinder DNA extraction and microbial and genetic target detection. Alternatively, the overrepresentation of mammals and birds may reflect historical research priorities and existing sampling infrastructure.

The limited number of studies on wild plants may reflect a perceived lower relevance of plant-associated AMR to human and livestock health. Unlike animals, wild plants are less genetically related to humans and are consumed less frequently than domesticated crops, reducing their perceived risk as vectors of human pathogens. When searching for publications on AMR in wild plants, studies on AMR in crop plants were identified. Although these studies were excluded from the database, crop-focused research far outnumbered studies on wild plants, likely because of their emphasis on fungal resistance to fungicide treatments, which carry significant food security implications. Unlike crops, wild plants are not consistently consumed, and foraging depends on species edibility and local practices. In addition, some wild plants are collected for traditional medicinal use, creating another potential route of human contact. Any plant consumed raw (whether cultivated or foraged) can serve as a route of human exposure to AMR through environmental bacteria, potentially leading to colonisation or infection^7^.

Future research should aim to balance breadth and relevance by reducing the current bias toward mammals and birds through the inclusion of amphibians, reptiles, fish, invertebrates, and wild plants, while also being cognisant of the need to prioritise species with strong human-environment interfaces, such as rodents, gulls, and waterfowl, that are likely to play key roles in AMR transmission. Expanding taxonomic coverage alongside strategic targeting will provide a more comprehensive and risk-informed understanding of AMR dynamics across ecosystems. Increasing taxonomic coverage will require innovative, ethical sampling strategies, such as non-invasive swabbing, environmental DNA (eDNA) collection, and opportunistic sampling from carcasses or faecal matter, to overcome logistical and ethical challenges associated with capturing rare or sensitive species. Notably, rationales for existing studies were often focused on environmental dissemination and anthropogenic spillover, with fewer addressing human exposure or methodological innovation, highlighting the need for future work, particularly on human exposure risks. By broadening the scope of surveillance, researchers can generate a more comprehensive picture of AMR ecology and improve risk assessments under a One Health framework.

Sample and analysis type are often determined by the study’s objectives, for example, whether investigating AMR diversity in zoonotic pathogens or targeting specific ARGs. These choices are further influenced by host taxa, habitat type, and practical constraints such as time, expertise, and budget. The frequent use of faecal and gut microbiome samples likely reflects the role of the gut as a reservoir for enteric pathogens (e.g., *E. coli*^87^, *Salmonella* spp^86^. and *Klebsiella* spp^46^.) and its dominance in the overall host microbiome^105^. Human and animal gut microbiomes are also considered as reaction vessels, where selection for and HGT of ARGs may occur^106^. Also, faecal sampling is popular due to its non-invasive nature. Other common non-destructive samples include nasal, oral, and oesophageal swabs, which target pathogens like *Staphylococcus* spp. Ultimately, sample selection depends on the research question and logistical constraints (e.g., time, environment type, expertise, and budget), a trend similarly noted in environmental AMR surveillance reviews and global AMR monitoring recommendations (e.g., Berendonk et al. (2015)^107^, Liguori et al. (2022)^108^, World Health Organisation (WHO) Global Tricycle Surveillance^109^).

AST and PCR were the most commonly used analytical methods in the literature, likely due to their long-standing use and accessibility. In contrast, other approaches, such as qPCR, metagenomics, and whole genome sequencing, were less frequently applied, possibly due to higher costs, specialised equipment, and technical expertise required for both execution and analysis. However, these approaches offer substantial benefits, as qPCR provides highly sensitive, accurate, and quantitative detection of specific ARGs, particularly of low‑abundance or dilute samples^110^; metagenomics enables broad, cultivation‑independent profiling of the resistome, capturing mobile genetic elements, and does not rely on a priori gene selection^110^; and whole genome sequencing offers high‑resolution insights into strain identity, genomic context of ARGs, and transmission pathways^111^. Both AST and PCR have limitations, as AST is restricted to culturable organisms, representing only a fraction of microbial diversity, while PCR requires a priori selection of gene targets. Among the studies that used AST and targeted culturable microorganisms, the most studied microbial targets included enteric bacteria (e.g., *Escherichia* spp., *Enterococcus* spp. and *Salmonella* spp.) and human pathogens (e.g., *Campylobacter* spp., *Pseudomonas* spp. and *Staphylococcus* spp.). Many of these genera contain zoonotic disease-causing species, that have been shown to spread easily through different environments, from animals to humans, and also carry AMR (e.g., resistant *Campylobacter jejuni*^74^), making them more likely to be a target of interest. Many of the bacterial genera frequently studied, such as *Salmonella* spp., *Pseudomonas aeruginosa*, and *Staphylococcus aureus*, are listed on the WHO Bacterial Priority Pathogens List due to their multidrug resistance and potential for rapid spread^112^. However, certain WHO Bacterial Priority Pathogens were absent from the reviewed studies, for example, *Neisseria gonorrhoeae*, which is unsurprising given its strict adaptation to humans, unlike other bacteria with environmental reservoirs or zoonotic hosts. This underscores the necessity of accounting for pathogen biology and epidemiology when interpreting gaps in wildlife AMR surveillance literature.

Studies often focused on specific species, rather than all culturable bacteria (a small proportion of all bacteria), contributing to taxonomic bias; a finding also highlighted by Torres et al., (2020)^26^. In part, these patterns likely reflect purposeful prioritisation, where many studies target enteric and other well-established clinically relevant indicator organisms (e.g., *E. coli*) and use culture-based AST methods because these approaches are comparatively rapid, inexpensive, and widely available across settings. We therefore interpret some microbial and methodological patterns not only as sources of knowledge gaps, but also as pragmatic choices that maximise comparability with clinical AMR frameworks and enable participation by research groups with diverse resources. In studies using genetic amplification methods (e.g., PCR, qPCR), the most frequently targeted genes conferred resistance to beta-lactams, tetracyclines, and aminoglycosides. with *bla*_*TEM*_, *tetA*, *bla*_*OXA*_, *bla*_*SHV*_, and *tetB* among the most common. However, when all *bla*_*CTX-M*_ variants were combined, this gene family emerged as the most frequently targeted, likely due to its clinical relevance as the most abundant ESBL group^113^. Many of the most frequently studied analytical targets (e.g., *E. coli*, *Pseudomonas aeruginosa*, *bla*_*CTX-M*_, *sul1*) align with those often suggested for environmental surveillance of AMR by, for example, Berendonk et al. (2015)^107^ and Liguori et al. (2022)^108^.

A notable bias in microbial targets was the distinct lack of fungal AMR studies in wildlife (2% of total publications). This gap likely stems from the perception that fungal resistance is primarily a concern for crop and human pathogens, with limited attention given to environmental niches. Furthermore, fungal resistance operates through mechanisms distinct from those of bacterial resistance, necessitating tailored research strategies rather than reliance on bacterial resistance paradigms. Some studies focused on wildlife health have examined fungal infections in poikilothermic animals (e.g., *Batrachochytrium dendrobatidis* infection in amphibians^114^), but these are often disconnected from human health relevance. Even environmental studies targeting fungal pathogens for human health rarely assess resistance, focusing instead on infection prevalence (e.g., Dutch elm disease caused by *Ophiostoma* spp.). This lack of emphasis on fungal AMR reflects broader trends in AMR research and policy, where fungi remain under-prioritised despite their significant health burden^115,116^. For example, *Cryptococcal meningitis* receives less than a quarter of the research funding as that of bacterial *Neisseria meningitidis*, yet is responsible for 20 times more deaths^117^. This is concerning, as fungal pathogens such as *Cryptococcus neoformans*, *Candida* spp., and *Aspergillus fumigatus* can be highly resistant and associated with high mortality rates^118^, and have been widely isolated from the environment (e.g., azole-resistant *A. fumigatus*^119^). Compounding this issue is the extensive use of azole antifungals in agriculture, which exerts strong selective pressure on environmental fungi, facilitating the emergence of resistant strains that can infect humans^120^. This plant-fungi-human interface underscores the need to consider environmental and agricultural practices in AMR surveillance. The current underreporting of antifungal resistance in wildlife leaves a critical gap in our understanding of its environmental dynamics, wildlife reservoirs and potential clinical relevance to human populations.

Future research directions should build on existing strengths while addressing current limitations. Culture-based methods and AST remain essential for phenotypic resistance profiling and understanding clinically relevant pathogens, but they capture only a fraction of microbial diversity. Combining these approaches with molecular techniques such as PCR, qPCR, whole genome sequencing (as is often done), and metagenomics will provide a more comprehensive view of AMR, including unculturable organisms and broader ARG diversity. Expanding metagenomic databases to include data from wild hosts is vital for capturing the full range of environmental and wildlife-associated AMR, especially in under-sampled taxa. Increasing the coverage will help to address current knowledge gaps, ensuring that surveillance efforts reflect true ecological diversity and risk across all wildlife. Expanding sampling to various tissues and environmental matrices, beyond faecal material to will further improve surveillance and help uncover environmental reservoirs. Importantly, fungal AMR in wildlife is critically underexplored despite its clinical importance, and should be prioritised alongside broader bacterial targets. However, it is important to recognise that clinical AMR is often driven by a small number of high-priority pathogens typically associated with healthcare transmission. The intention is not to suggest that wildlife or environmental reservoirs are the main sources of AMR, but rather to underline their role as sentinels and supplementary surveillance targets at the convergence of human, animal, and environmental systems. Monitoring these reservoirs can help identify the emergence and transmission of AMR, which may be significant locally and inform policymaking. This perspective highlights the interconnected nature of AMR dynamics and the importance of environmental surveillance within a One Health strategy. Finally, integrating these efforts within a One Health framework and leveraging sentinel species will enable more effective monitoring of anthropogenic AMR spillover and inform targeted interventions, ultimately improving our understanding of resistance across ecosystems and its implications for public health.

This semi-systematic review collated global data on AMR in wild animals and plants. The resulting database (provided as a Supplementary File) serves as a valuable resource for both researchers and policymakers. The findings indicate growing interest in this field, yet also reveal significant biases, particularly the underrepresentation of certain countries and host taxa. The identified trends and knowledge gaps, particularly the scarcity of research into fungal AMR, as well as wild amphibians and plants, can inform and shape future research priorities and funding strategies. Rationale analyses revealed that most studies focused narrowly on environmental dissemination and anthropogenic spillover, with fewer addressing human exposure, aligning with a broader lack of integrated studies that apply a One Health approach to connect human, environmental, and wildlife niches. Such integration is essential to move beyond fragmented objectives and improve our understanding of pressure-response dynamics and the drivers of AMR transmission across interconnected systems. To enable widespread adoption of wildlife-based AMR surveillance, standardised methodologies across countries and environmental and taxonomic contexts are needed to ensure data comparability. If achieved, wildlife could serve as robust indicators of environmental AMR pressures, as their microbiomes reflect surrounding anthropogenic influences. Surveillance of this type could inform both environmental health monitoring and risk assessments for human and livestock health, particularly in shared environments where interactions between humans, animals, and wildlife are common.

## Methods

### Literature searching

This semi‑systematic review utilised a literature database originally compiled for an Environment Agency report^29^. Briefly, the methodology used for the report included semi-systematic searching using Web of Science (WoS) (search query weblink here), supplemented with Google Scholar searches and backward citation chasing. Google Scholar searches helped to better capture plant‑based and antifungal studies, which were underrepresented initially. For the present study, we conducted new searches to supplement the previous database with additional and more recent studies. Literature searches were conducted using Web of Science, but with a revised and optimised search string. The search string used in WoS was: (TS = (AMR OR “anti* resistan*“)) AND (TS = (“Wildlife” OR (“wild” AND (“plant*“ OR “animal*“ OR “mammal*“ OR “bird*“ OR “reptile*“ OR “amphibian*“ OR “invertebrate*“ OR “fish*“)))) (see here for the saved query weblink). WoS searches were restricted to the “Topic” field, which searches titles, abstracts, authors, keywords and KeyWords Plus. To ensure our search terms were sufficiently comprehensive and sensitive, we cross-checked the results against a list of known, relevant papers in the field, verifying that these key publications were captured. This approach helped confirm that the search strategy was robust and unlikely to miss important studies. All search results for the refined search are up to date as of 23rd August 2023, after which point no more searches were performed.

### Literature screening

WoS search results were deduplicated and screened at title and abstract level by HT, RP and IS, with a minimum of 10% of articles double-screened by two authors. Only publications focusing on wild animals or plants were included in the database. Review articles were excluded. Other exclusion criteria included focus on food-producing animals (e.g., livestock and aquaculture) and plants (e.g., food crops and fruit trees), companion animals, and captive animals (e.g., those in zoos, zoological collections, and semi-managed populations in reserves). Wild animals in rehabilitation or rescue centres were included in instances where samples were taken from the wildlife upon arrival at the centre, prior to receiving any treatment or interference. Relevant publications where the full text was inaccessible were not included in the database.

### Database creation

Data from all relevant search results were extracted and collated in bespoke data extraction sheets. The extracted variables included summary data, methodological data, and rationale behind the study. The country of study (i.e., where samples originated from, not the country the authors were based in) was extracted.

Specifically, database column headings were as follows:

- Database ID,
- Author(s),
- Title,
- Published date,
- Citation,
- Study location (by country),
- AMR type (categories – Antibacterial or Antifungal),
- Organism type 1 (main categories – Animal or Plant),
- Organism type 2 (subcategories – Amphibian, Bird, Fish, Invertebrate, Mammal, Plant, Reptile),
- Organism 3 (host species),
- Environment type (categories – Coastal, Freshwater, Marine, Terrestrial),
- Sample (physical sample type, for example, faeces or oral swabs),
- Method type 1 (main categories – Culture-based or Molecular-based),
- Method type 2 (sample analysis type, for example, antibiotic susceptibility testing (AST) or quantitative polymerase chain reaction (qPCR). Only AMR methods were included),
- Culture target (microbes targeted for culture-based methods),
- Molecular target (antimicrobial resistance genes targeted for molecular-based methods), and
- Rationale (categories – Dissemination of AMR in the environment, Human health/Exposure to AMR, Spillover to the environment from anthropogenic contact, Testing methods or Other).

### Data analysis

Data were analysed for trends in country, region, environment type of study, publication date, target host taxa, analytical methods, sample types, culture and molecular targets, and study rationale. Data analyses were performed in Microsoft Excel and RStudio, and figures were created using the “ggplot2” package in R^121^ and ArcMAP v10.8.2 software^122^.

Target host taxa were extracted from each publication and categorised taxonomically. Where multiple host taxa appeared in each publication, the total number of occurrences for each taxon was counted within the database. Where lower taxonomic information was not given in the publication, taxa were recorded to the next best taxonomic level. For analyses, host taxa were grouped taxonomically at order level. Following this, some taxa were grouped at other taxonomic levels to better extract trends from the data. For example, the large order Artiodactyla (the even-toed ungulates) was split into families, such as *Bovidae* (cattle, etc.), *Cervidae* (deer, etc.), *Suidae* (pigs, etc.), *Camelidae* (camels, etc.) and more. This was also done for other organism types, such as birds, for the diverse order Charadriiformes, which was split into the suborders Charadrii (wading shorebirds) and Lari (gulls, etc.). Environment types were designated based on the habitat of the target host organism, with coastal and marine designations based on definitions described in Carrasco De La Cruz (2021)^123^.

Culture targets (bacteria and fungi) were extracted from each publication and categorised taxonomically to order, family, genera and even species level, whereas molecular targets (i.e., resistance genes) were extracted from publications and categorised according to antimicrobial class. As with target host taxa, multiple culture and molecular targets appeared in each publication, therefore, the total number of occurrences for each culture taxa or resistance gene were counted within the database.

## Supplementary information

Supplementary Information

Data S1

## Data Availability

All data generated or analysed during this study are included in this published article and its supplementary information files.
