# Supplementary material for: AMR in wild animals and plants: global trends and future priorities in wildlife-associated antimicrobial resistance research
Source: NPJ Antimicrob Resist. 2026 Jun 2;4:48. doi: 10.1038/s44259-026-00226-3 (PMC13518989; doi:10.1038/s44259-026-00226-3)
Supplement: Supplementary file 1 [file 44259_2026_226_MOESM1_ESM.pdf]

AMR in wild animals and plants: Global trends and future priorities in wildlife-associated antimicrobial resistance research

Authors:

Holly J. Tipper[1*#], Rachel A. Payne[1#], Isobel C. Stanton[1], Jennifer M. G. Shelton[1], Alwyn Hart[2], Wiebke Schmidt[2], Purvi Mali[2], Andrew C. Singer[1], Daniel S. Read[1*]

Affiliations:

1. UK Centre for Ecology & Hydrology (UKCEH), Wallingford, Oxfordshire, UK, OX10 8BB.

2a. Chief Scientist's Group, Environment Agency, Horizon House, Deanery Road, Bristol, UK, BS1 5AH.

2b. Formerly Chief Scientist's Group, Environment Agency, Horizon House, Deanery Road, Bristol, UK, BS1 5AH.

* Corresponding authors: Holly J. Tipper, email address: holtip@ceh.ac.uk; Daniel Read, email address: daniel.read@ceh.ac.uk

[#] Joint first authors

Supplementary information

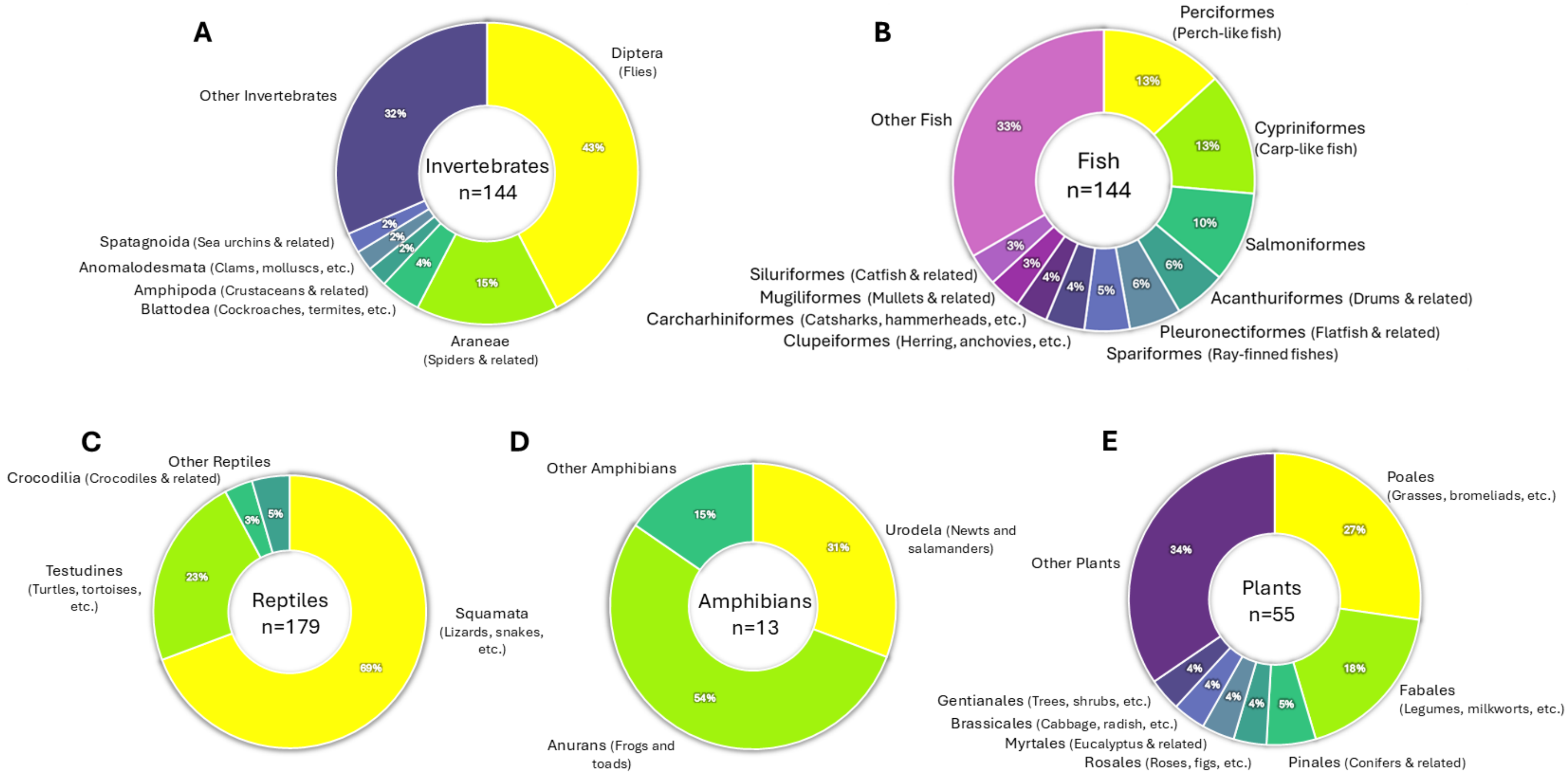

Figure S1. Proportion of different types of a) invertebrates (49 publications), b) fish (34 publications), c) reptiles (59 publications), d) amphibians (10 publications) and e) plants (14 publications). N = total occurences within the set of publications for each group; percentages indicate percentage of total occurrences.

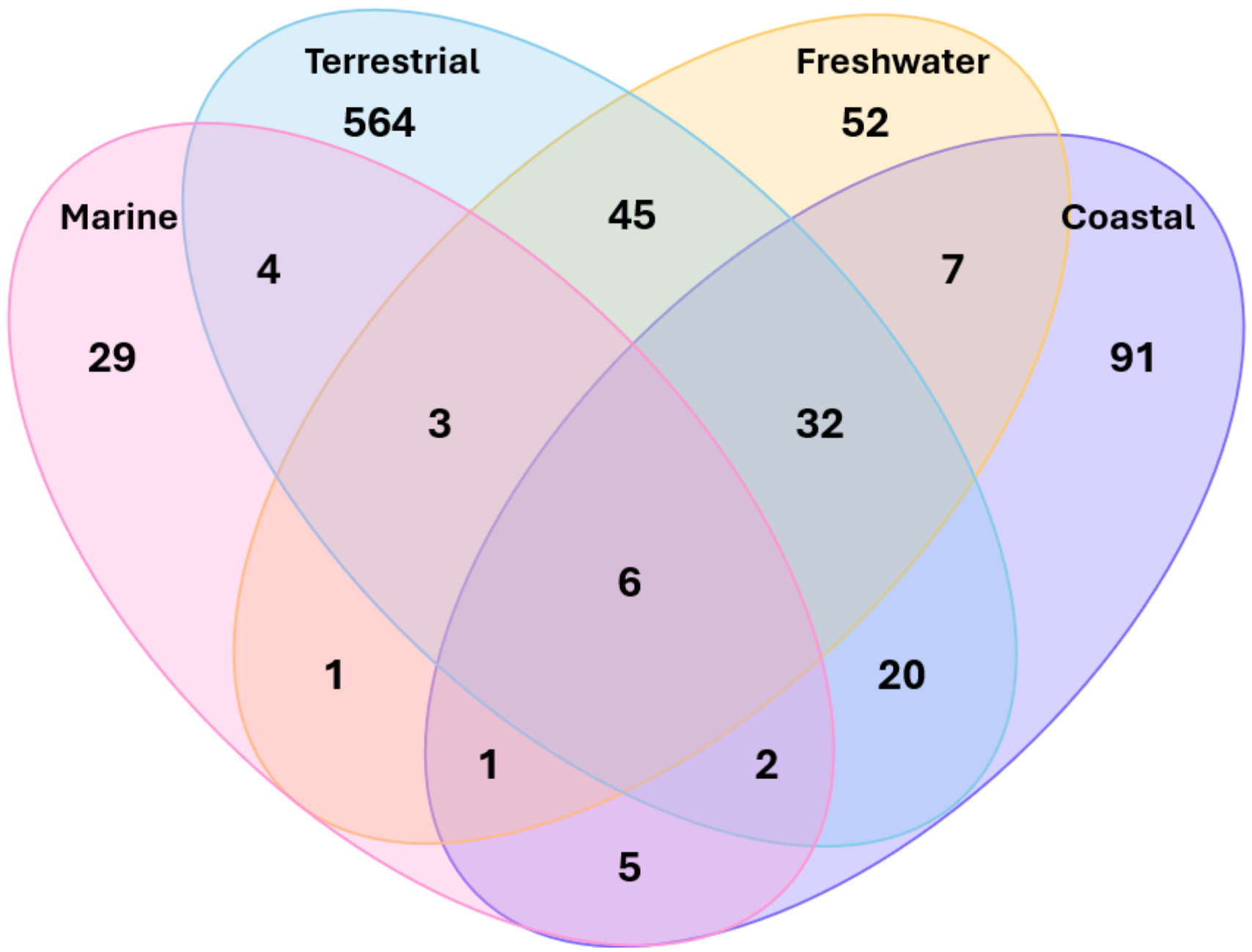

Figure S2. Publications containing environment types of sampled organisms. Organisms were split into Marine, Terrestrial, Freshwater, and Coastal environments. Overlaps show where publications sampled multiple organisms which came from different environment types. Six publications (not shown here) did not include organism detail to a level where a habitat could be assigned, so were defined as ‘unknown’.

Data S1. Database of publications on AMR in wild animals and plants, compiled from semi-systematic searches. Updated to 23 August 2023.
